# Supplementation with willow containing condensed tannins shifted nitrogen excretion from urine to faeces in yearling ewes

**DOI:** 10.1016/j.animal.2025.101698

**Published:** 2025-12

**Authors:** J.P. Thompson, O. Cristobal-Carballo, T. Yan, W.E. Zeller, S. Huws, L. Safoi, A.D. Southam, C. Ludwig, G.R. Lloyd, S. Stergiadis, K. Theodoridou

**Affiliations:** a Institute of Global Food Security, Queen’s University Belfast, Belfast, UK; b School of Biological Sciences, Queen’s University, Belfast, UK; c Sustainable Livestock Systems Branch, Agri-Food and Biosciences Institute, Hillsborough, UK; d School of Agriculture, Policy and Development, University of Reading, Reading, Berkshire, UK; e USDA-ARS, U.S. Dairy Forage Research Center, Madison, WI, USA; f Phenome Centre Birmingham, School of Biosciences, University of Birmingham, Edgbaston, Birmingham, UK; g Department of Metabolism and Systems Sciences (MSS), College of Medicine and Health, University of Birmingham, West Midlands, UK

**Keywords:** Bioactive compounds, Digestibility, Energy, Metabolites, Sustainability

## Abstract

Ruminants have poor nitrogen use efficiency, with 70% of total ingested nitrogen (**N**) excreted. Much of this excreted N is in the form of urinary urea which is easily hydrolysed to ammonia. Increasing ammonia production has negative impacts on environmental and human health. Dietary supplementation with condensed tannins (**CTs**) could be a solution as they bind to the N source (protein) in the rumen, preventing degradation, allowing more N to reach the abomasum for digestion and absorption. However, if this N is not absorbed, it results in a shift in N excretion from urine to faeces. The aim of this study was to evaluate the effect of willow containing CTs through a 21% DM dietary inclusion of two willow varieties (*Salix Beagle*: **BG** and *Salix Terra Nova*: **TN**) mixed with grass silage. Willow treatments: BG and TN had a CT concentration of 1.20 and 0.08% DM, with the control (**SIL**) containing no CTs. Twelve replacement yearling ewes were used in a 3 (treatment) × 3 (period) Latin square design experiment with a single period lasting 28 days and the overall experiment lasting 12 weeks. Structural arrangement of CTs showed BG had a greater (*P* < 0.001) proportion of prodelphinidin (62.7%) compared to TN (5.42%). The higher CT-containing treatment (BG) showed a 13 and 12% lower absorbed N-to-N intake ratio relative to SIL (*P* < 0.001) and TN (*P* < 0.01), respectively. There were no differences in the proportion of retained N to N intake among feed treatments (*P* = 0.44). BG had a 22 and 17% greater faecal N to N intake ratio relative to SIL (*P* < 0.001) and TN (*P* < 0.01), respectively. BG led to a 19% higher nitrogen excretion in faeces and a 19% lower nitrogen excretion in the urinary form compared to SIL (*P* < 0.01). However, BG reduced (*P* < 0.01) the nutrient digestibility, compared to SIL and TN. This resulted in the ratio of digestible energy to gross energy intake to be 8 and 6% lower for BG relative to SIL (*P* < 0.01) and TN (*P* < 0.01), respectively. Feed treatments had an effect on blood and urine metabolites involved in protein metabolism. Isobutyrate was 17 and 28% higher in BG and TN relative to SIL (*P* < 0.01). While in urine, creatinine was 61% lower in TN relative to BG (*P* < 0.05). CTs from the willow Beagle variety could redirect nitrogen excretion from urine to faeces, offering a promising strategy to reduce ammonia emissions and mitigate the environmental footprint of the ruminant industry.

## Highlights

• Our study evaluated 21% dietary inclusion of two willow varieties in ewe diets.
• Condensed tannin structure affects nutrient binding and digestibility in ruminants.
• Beagle variety reduced the digestibility of energy, fibre, and protein.
• Beagle variety shifted nitrogen excretion from urine to faeces.
• Willow may help reduce the environmental footprint of ruminant production.

## Implications

Ruminants poorly utilise nitrogen, which leads to increased ammonia production with negative impacts on the environment and human health. The aim of this study was to evaluate the effect of feeding ruminants willow containing condensed tannins on nitrogen use efficiency. Tannins from the Beagle willow variety reduced nitrogen in urine and increased nitrogen in faeces, where it is in a more stable and environmentally friendly form. Willow may provide a useful strategy to reduce ammonia emissions and help lower the environmental footprint of the ruminant industry.

## Introduction

The global ruminant industry is continuing to grow, mainly driven by increasing consumer demand for dairy and red meat in developing countries (Pulina et al., 2021). However, ruminant animals have a much lower feed efficiency than monogastric animals, leading to higher reactive nitrogen and greenhouse gas (**GHG**) emissions per unit of ruminant protein production (Cheng et al., 2022). While ruminants have a unique ability to convert fibrous plant material (indigestible by humans) into energy, improvements in energy and nitrogen use efficiency (**NUE**) are being pursued to reduce environmental impacts and improve economic viability of the industry (Huws et al., 2018).

Previous studies showed that CH_4_ produced by ruminants represents a 2–8% loss of dietary energy for the ruminant and in some cases, up to 12% where feed quality is low (Johnson and Johnson, 1995). Therefore, reducing ruminal CH_4_ production by ruminants has the potential to increase energy use efficiency and reduce GHG emissions. Also, ruminants are poor utilisers of nitrogen (**N**) with approximately 70% of ingested N excreted (MacRae and Ulyatt, 1974). Much of the excreted N is in the form of urinary urea which is rapidly hydrolysed to ammonia (NH_3_) by urease enzyme present in both faeces and soil (Voorburg and Kroodsma, 1992). Between 1980 and 2018, NH_3_ emissions from livestock production increased by 45%, with ruminant production being a major contributor to this rise (Liu et al., 2022). Consequently, strategies to enhance the NUE of ruminants have focused on reducing the overall nitrogen content in their diet or utilising bioactive compounds to manipulate rumen fermentation, enhance digestibility, and increase productivity. There is sufficient published literature to suggest that condensed tannins (**CTs**) are bioactive compounds that have potential to modify rumen fermentation, dietary utilisation and animal performance when provided in the diet. It is well known that CTs have a mitigating effect on ruminal CH_4_ production, with one study reporting a 34% reduction when cattle grazed a *Lotus corniculatus* sward of CT inclusion of 8% DM (Woodward et al., 1999). This reduction in CH_4_ has the potential to increase metabolisable energy. Regarding NUE, it is well known that CTs can bind protein in the rumen, forming a complex that prevents degradation (Barry et al., 2001). As the complex passes from the rumen to the abomasum, the pH drop causes the dissociation of the CT and protein, releasing more true protein into the hindgut, increasing the potential for greater N uptake for maintenance, growth, production and other metabolic functions (Singh and Bhat, 2001). If nitrogen is not absorbed in the small intestine, it may lead to a shift in nitrogen excretion from urine to faeces, which represents a more environmentally stable form of nitrogen. A previous study showed that CTs induce a 9.3% reduction in urinary N as a proportion of total N losses (Carulla et al., 2005), while another study showed a 25% reduction (Misselbrook et al., 2005). Furthermore, another feeding trial on high-producing dairy cows, using quebracho tannin extract at levels ranging from 0 to 1.8% (DM basis), observed a linear decrease in urinary nitrogen accompanied by a linear increase in faecal nitrogen excretion (Duval et al., 2016). In that case, NH_3_ emissions from the slurry of those animals were between 8 and 49% less than the emissions from the control slurry (Duval et al., 2016).

Willow (*Salix* sp.) fodder is used in biofuel production, but leaves and branches up to 18 mm in diameter are not used from these harvests, and constitute a considerable waste stream (Ortuño et al., 2021). This willow tree fodder stream contains CTs, which could have the potential to improve energy and NUE in ruminants. Nevertheless, there is a wide variation in the mode of action of CTs in its ability to bind to nitrogen and fibre in addition to inhibiting methanogenesis between sources. Structural components such as: mean degree of polymerisation (**mDP**), ratio of procyanidins to prodelphinidins (**PC:PD**) and ratio of *cis* and *trans* have been shown to affect the bonding capacity of the CTs (Huyen et al., 2016). The strength of the CT binding capacity has been linked positively to mDP and prodelphinidin content due to the greater number of flavan-3-ol subunits and a greater number of sites for hydrogen bonding to occur (Gea et al., 2011). Although structural assessments of CTs are well explored, the structure of willow CTs is underexplored.

This study aimed to explore, a) the effects of structure, content and biological activity of CTs in leaves of two willow (*Salix*) varieties on *in vivo* nutrient digestibility, energy and NUE of growing female sheep (yearling ewes) and b) assess their impact on metabolic pathways and production-related processes in digestion by analysing blood and urine metabolites. This novel data will enable us to determine the impact of CTs from these willow varieties on ruminants' nitrogen metabolism, deposition, and excretion pathways.

## Material and methods

### Animals and housing

The experimental research study occurred at the Agri-Food and Biosciences Institute, Northern Ireland (54.45° N, 6.07° W, and 112 m altitude). Twelve replacement yearling ewes (6 Texel × mule and 6 Suffolk × mule) were used in a 3 (treatment) × 3 (period) Latin square design experiment with a single period lasting 28 days and the overall experiment lasting 12 weeks. Yearling ewes aged approximately 13 months and weighing an average of 66.63 kg (s.d. 6.17 kg) were allocated to three treatment diets balanced according to sire breed, dam breed and BW. Each treatment group was composed of 2 Texel × mule and 2 Suffolk × mule yearling ewes. Throughout the experiment, during each period, the first 3 weeks, yearling ewes were housed in a single group on plastic slats with access to water and treatment diets through the biocontrol feeding system. During this phase, the initial 3 weeks were counted as the adaptation phase. Subsequently, on the first day of week 4, yearling ewes were transferred to individual metabolic crates for a week of digestibility measurements. Each crate contained a feed bin, drinking water container and separate trays to collect faeces and urine.

### Willow fodder harvesting

Two varieties of willow, Salix Beagle (**BG**) and Salix Terra Nova (**TN**), were selected for experimentation based on condensed tannin contents and palatability. Willow fodder consisted of leaves only and harvesting commenced in early May 2022 until mid-July 2022. Fodder was stripped from willow branches by hand and chopped (up to 4 cm) using a BOSCH AXT 25 D 2500W Shredder (BOSCH, Milton Keynes, United Kingdom). Weighed amounts of willow fodder according to daily DM requirements were compressed into a plastic bag. Anaerobic conditions were created using a Delfin 202 DS Industrial vacuum (Delfin, Italy, Europe) to draw out air with the bag fully sealed. Each fodder bag was spot-frozen at −80 °C for 5 days and then moved to a walk-in freezer at −20 °C for the remainder of storage. Each bag was removed 24 h before feeding and allowed to defrost in a fridge at 3–5 °C.

### Treatments

During a 2-week pre-experimental period, yearling ewes were offered grass silage (**SIL**) plus the same concentrate (0.18 kg) administered during the experiment through the GreenFeed system (C-lock, South Dakota, USA). During the experiment, all yearling ewes were fed *ad libitum*. Treatments examined (SIL, BG and TN) differed in willow fodder inclusion and tested two different willow varieties. The BG and TN diet consisted of 20% willow fodder and 80% grass silage on a DM basis and were weighed out accordingly and mixed using a Belle Premier 200XT mixer (Belle, Derbyshire, United Kingdom). The SIL diet consisted of 100% grass silage and was used as the control. All treatments were supplemented with 0.18 kg of concentrate (Intensive lamb pellet, Fane Valley Feeds, Northern Ireland; Supplementary Table S1).

### Nutrient utilisation measurement

In the digestibility crates, feeding occurred daily at 0900 h with yearling ewes fed according to allocated treatment and a set amount of concentrate. Treatments, uneaten, were removed from each crate and weighed the following day at 0800 h. Quantities of dietary treatment offered and refused were recorded daily for each yearling ewe and samples of treatment forage offered and refused were retained daily for measurement of DM concentration at 85 °C for 24 h. Yearling ewes had constant access to water with the container attached to the digestion crate. BW was measured before entering and leaving the digestibility crate. While the yearling ewes were in the digestion crates for 7 days, nutrient utilisation measurement commenced 24 h after yearling ewes were moved to the crates, comprising a 6-day measurement period. Upon defecation and urination, the crate was designed to separate the two, allowing faeces to be collected in a plastic collection tray (50 × 100 cm) while the urine was collected into a separate plastic container (30 × 30 cm). To minimise N losses as ammonia, 10 mL of 50% sulphuric acid was added to each urine container daily. The total weight of faeces and urine produced during each 24 h collection period was recorded, and a sample of each (0.05 kg by weight) was stored in a fridge at 4 °C until the final day of collection, when the six daily faecal and urine samples were bulked for subsequent analysis. Fresh faeces and urine were analysed for N and gross energy (**GE**) content. Faeces samples were dried at 60 °C for 100 h to determine DM content and then milled to analyse ADF, NDF, ash and GE content.

Nitrogen intake (NI), faecal nitrogen (N) output, and urinary N output were measured to assess nitrogen utilisation. Absorbed N was calculated as the difference between nitrogen intake and faecal N output:

(1) Absorbed N (g/day) = NI-Faeces N

Retained N was determined by subtracting total nitrogen losses (faeces and urine) from the nitrogen intake:

(2) Retained N (g/day) = NI – (Faeces N + Urine N)

These calculations were used to evaluate nitrogen absorption efficiency and retention in the animals.

### Feed sampling and analysis

Daily samples of each diet fed (SIL, BG and TN) were collected and bulked together for a weekly representation of diet in the digestibility crates. Sample collection, preparation and analysis for DM, ether extract, ash, ADF, NDF, nitrogen content, CP, starch and GE were determined as outlined in Thompson et al. (2025). As also detailed in Thompson et al. (2025), an additional weekly fresh sample of the control, in this case grass silage, was analysed using near infrared spectroscopy for DM, CP, ADF, NDF, water soluble sugars (%DM) and metabolisable energy (ME; MJ/kg DM).

Willow ME was calculated using an *in vitro* model based on the level of gas production from the chemical composition of the willow (Menke and Steingass, 1988).

ME(MJ/kgDM)=2.00+0.1298GP+0.0045CP+0.0303Fat

where GP = gas production incubated for 24 h (ml/200 mg), CP = willow CP content (g/kg DM) = willow fat content (g/kgDM).

### Condensed tannins

Weekly forage samples were collected for the determination of their bioactive compounds (condensed tannins) from three different collection points, pooled and oven-dried at 30 °C for 96 h, passed through a 1 mm sieve and stored in a dark place. The *Salix* Beagle CT reference standard was obtained following the procedures described in Brown et al. and Naumann et al. (Brown et al., 2017, Naumann et al., 2018) and analogous to the procedures provided in Thompson et al., 2025, using the following quantities: extraction solvent (3 × 7:3 acetone/water, 500 mL); amount of ethyl acetate used to extract non-polar organic, 1st extraction 400 mL, 2nd extraction 450 mL; material amount of initial dried extract obtained, 11.31 g; volume of 1:1 methanol/water used to dissolve extract (100 mL); Sephadex LH-20 mass used, 33.30 g; 1:1 methanol/water washes of Sephadex LH-20 (15 × 200 mL); 7:3 acetone/water washes of Sephadex LH-20 (4 × 200 mL); mass of freeze-dried, purified CT from *Salix* Beagle sample, 1.79 g of an off-white solid, sufficiently pure to serve as the *Salix* Beagle CT reference standard in this study.

The CT used for the nuclear magnetic resonance (NMR) sample for *Salix* Terra Nova sample was obtained in a similar manner with the following quantities: extraction solvent (3 × 7:3 acetone/water, 500 mL); amount of ethyl acetate used to extract non-polar organic, 1st extraction 600 mL, 2nd extraction 300 mL; material amount of initial dried extract obtained, 10.0 g; volume of 1:1 methanol/water used to dissolve extract (100 mL); Sephadex LH-20 mass used, 39.07 g; 1:1 methanol/water washes of Sephadex LH-20 (15 × 100 mL); 7:3 acetone/water washes of Sephadex LH-20 (4 × 100 mL); mass of freeze-dried, purified CT from *Salix* Terra Nova sample 2, 0.032 g (32 mg). Two-dimensional NMR spectroscopy of these samples was conducted as described in Thompson et al. (2025).

For sequential CT content determinations, the HCl-butanol-acetone-iron assay was used (Grabber and Zeller, 2019) and is described in detail in Thompson et al. (2025).

### Metabolomic analysis by nuclear magnetic resonance

Metabolites were extracted from serum samples using a modified version of a biphasic methanol/chloroform/water method we have used previously (Southam et al., 2020). Briefly, 350 µL of serum was mixed with 788 µL ice-cold methanol (LC-MS grade, VWR), 150 µL ice-cold chloroform (HPLC plus grade, Sigma) and 315 µL ice-cold water (LC-MS grade, VWR), and then, the sample was vortexed (full power, room temperature [ca. 20 °C], 30 s). Samples were centrifuged (2 500 g, 18 °C, 10 min), and 1 047 µL of supernatant was removed and mixed with 500 µL chloroform and 313 µL water. Samples were vortexed (full power, room temperature [ca. 20 °C], 30 s), centrifuged (2 500 g, 18 °C, 10 min) and left for 5 min at room temperature [ca. 20 °C] to allow completion of the biphasic separation. Then, 1 mL of the upper phase (containing the polar metabolites) was removed into a 2 mL microfuge tube (Eppendorf) and dried in a SpeedVac concentrator (Savant SPD111V230, Thermo Fisher Scientific). Dried samples were stored at −80 °C until NMR analysis. Prior to NMR analysis, serum samples were resuspended: in 700 µL 10:90 Urine Preparation NMR Buffer (Avance IVDr, Bruker):LC-MS grade water with formic acid (0.5 mM). Samples were left at room temperature for 15 min and then centrifuged (20 000 g, 20 °C, 15 min), and 600 µL was loaded into a 5 mm NMR Tube (Bruker).

Regarding urine preparation, 750 µL of urine was centrifuged (20 000 g, 20 °C, 15 min) and 630 µL supernatant removed to a clean 2 mL microfuge tube (Eppendorf). 70 µL Urine Preparation NMR Buffer (Avance IVDr, Bruker) was added, and the sample was vortexed (full power, room temperature, 15 s), incubated at room temperature (15 min) then centrifuged (20 000 g, 20 °C, 15 min), and 600 µL was loaded into a 5 mm NMR Tube (Bruker).

NMR spectra were acquired on a Bruker IVDr 600 MHz spectrometer equipped with a 5 mm inverse probe. The water resonance was suppressed using a NOESY presat pulse sequence. A total of 128 transients were acquired after eight steady-state scans. The interscan relaxation delay was set to 10 s. Before acquisition, each sample was shimmed to a TMSP linewidth less than 1 Hz. The resulting NMR spectra were processed and prepared for statistical data analysis using MetaboLabPy (version 0.9.29 (Ludwig, 2025)). Each free induction decay was exponentially line-broadened by 0.3 Hz and zero-filled to 131 072 data points before Fourier transformation. All spectra were then manually phase corrected. To prepare the NMR spectra for statistical data analysis, the left and right edges and the water region were excluded. A total of 32 areas were segmentally aligned. Noise filtering was applied to exclude data points below five times the SD of the spectral noise.

The Chenomx software (Chenomx Inc., version 8) was used to determine metabolite concentrations. The metabolite concentration matrix from Chenomx was auto−scaled before 26 blood and metabolites and 20 urine metabolites were selected based on metabolic pathways that could be affected by CT inclusion in the diet (Saleem et al., 2012, Zhang et al., 2022, Kim et al., 2023, Mohammadi et al., 2023). These selected metabolites undertook multivariate analysis.

### Calculations and statistical analysis

All statistical analyses of data in the study were performed using R studio version 4.3 (R studio, Boston, MA, USA). All results were assessed for residual normality and homogeneity of variance and subsequently analysed using the ANOVA or Kruskal-Wallis test.

ANOVA:

$$\mathrm{Yij}=\mu \;+Si\;+Ij\;+\left(SI\right)ij\;+Eij$$

where *Yij* is the dependent, continuous variable; *µ* is the overall mean; *Si* and *Ij* are the fixed effects of feed treatment, respectively; (SI)ij is the interaction of the effect and *Eij* is a residual error.

Kruskal-Wallis:

$$H=\frac{12}{N\left(N+1\right)}\times \left[\sum_{j=1}^{k}\frac{{\mathrm{Rj}}^{2}}{\mathrm{nj}}\right]-\;3\left(N+1\right)$$

where *N* = total number of observations across all groups; *k* is the number of groups; *Rj* is the sum of ranks for group *j*; *nj* is the number of observations in group *j*. Differences between treatments were considered significant when *P* < 0.05 following posthoc tests using the pairwise *t*-test.

Regarding selected metabolite concentrations, multivariate analysis using principal component analysis (Wold et al., 1987) and partial least squares discriminant analysis (Wold et al., 2001) were applied to the selected metabolites using the mixOmics package (version 6.28.0) (Lê Cao et al., 2009). Hierarchical cluster analysis between feed treatments using average linkage and the Euclidean distance metric was visualised using the pheatmap package (version 1.0.12) (Kolde, 2019). Annotation of the class of metabolite (e.g. Protein, carbohydrate and fatty acid digestion) based on its involvement in digestive pathways was adapted from the Kyoto encyclopaedia of genes and genomes database (Supplementary Table S2).

Methods for calculating the structural features of CTs are described in Zeller et al. (2015) (*cis*/*trans* and PC/PD ratios) and Naumann et al. (2018) (mean degree of polymerisation, mDP).

## Results

### Condensed tannin intake and structure differ between willow treatments

**Fig. 1:**
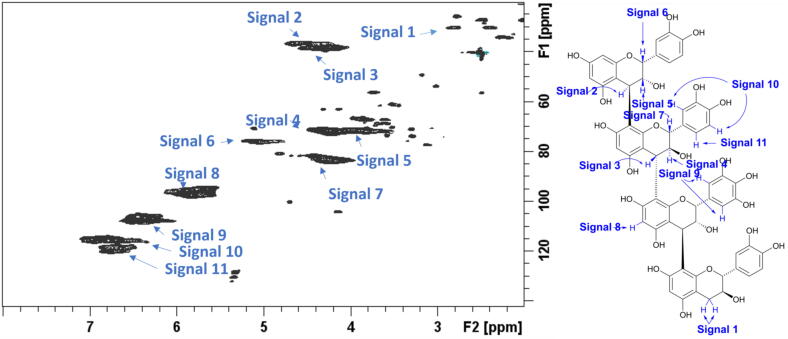
^1^H–^13^C Heteronuclear Single Quantum Coherence Nuclear Magnetic Resonance spectrum of the Salix Beagle Condensed Tannins with signals identified as listed in the accompanying condensed tannin structure, analysed in samples offered to ewes.

**Fig. 2:**
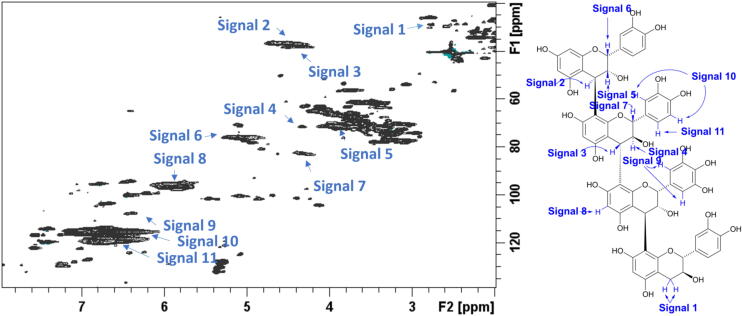
^1^H–^13^C Heteronuclear Single Quantum Coherence Nuclear Magnetic Resonance spectrum of the Salix Terra Nova Condensed Tannins with signals identified as listed in the accompanying condensed structure, analysed in samples offered to ewes.

The determination of unbound, bound and total CT content is displayed in Table 1. In this study, we used the unbound CT content for forage and formulated diet (Supplementary Table S3) CT content. Unbound CTs are free to react and influence feed particles or the rumen microbiome. For BG and TN, the diets consisted of 13.46 and 0.86 g CT/kg DM while no CT were detected in SIL. The two-dimensional (2D) ^1^H-^13^C Heteronuclear Single Quantum Coherence (HSQC) Nuclear Magnetic Resonance (NMR) spectra of the purified CTs from samples *Salix* Beagle and *Salix* Terra Nova are displayed in Fig. 1, Fig. 2 respectively, with Table 1 displaying the structural determination data extracted from the NMR spectra. Three contrasting features are evident in the CT structural arrangement. First, BG CT had an average *cis/trans* ratio of approximately 1:4, whereas the average *cis/trans* ratio of TN CT was about 2:1 (*P* < 0.001). Second, the average PC/PD ratio for BG was approximately 1:2, whereas the average PC/PD ratio for TN was approximately 95:5 (*P* < 0.001). Thirdly, mDP was 1.56 times greater than BG relative to TN, but this difference was non-significant (*P* = 0.224).

**Table 1:** Condensed tannin content of the two willow varieties used in ewe diets as a percentage of total DM.

Item	SIL	BG	TN	SEM	*P*-value
Content					
Bound (%DM)	Nd	2.84	1.26	0.248	***
Unbound (%DM)	Nd	6.38	0.41	0.936	**
Total (%DM)	Nd	9.05	1.65	1.16	**
Structure					

mDP	Nd	7.31	4.66	1.185	0.224
%PC	Nd	37.30	94.58	7.001	***
%PD	Nd	62.70	5.42	7.001	***
Cis	Nd	19.98	63.96	5.383	***
Trans	Nd	80.02	36.04	5.383	***

SIL, silage; BG, Beagle willow variety; TN, Terra Nova willow variety; mDP, mean degree of polymerisation; PC, procyanidin; PD, prodelphinidin. The symbols, ‘**’,’**’, denote significance at *P* < 0.01 and 0.001, respectively.

Due to differences in CT content, CT intake (CTI) was affected by feed treatment (*P* < 0.001; Table 2). Ewe yearling ewes fed BG had a CTI of 17.50 g/d compared to 1.16 g/d for TN. Overall CT inclusion (%TDMI) was 1.20 for BG and 0.08 for TN (Table 2).

**Table 2:** Nutrient intake and Apparent Total Tract Digestibility of yearling ewes fed different feed treatments with differing condensed tannin inclusion.

	Treatment				
Item	SIL	BG	TN	SEM	*P*-value
Liveweight (LW)	65.6	67.6	66.6	1.04	0.670
Feed intake					
Willow inclusion (%DM)	0.00^a^	21.1^b^	21.0^b^	0.0170	***
Forage DMI (kg/d)	1.19	1.30	1.34	0.0237	0.0825
Concentrate DMI (kg/d)	0.161	0.161	0.161	−	−
Total DMI (kg/d)	1.35	1.46	1.50	0.0237	0.0772
OMI (kg/d)	1.22	1.33	1.36	0.0213	0.0676
NDFI (kg/d)	0.683	0.694	0.744	0.0119	0.189
ADFI (kg/d)	0.443^a^	0.476^ab^	0.519^b^	0.0101	*
CTI (g/d)	0.00^a^	17.5^b^	1.16^c^	1.36	***
CT inclusion (% TDMI)	0.00^a^	1.20^b^	0.0768^c^	0.0928	***
DMI/LW (kg/kg)	0.0207	0.0217	0.0226	0.000405	0.0896

Energy intake and use efficiency					
GEI (MJ/d)	26.0^a^	28.3^b^	29.2^b^	0.458	*
DEI (MJ/d)	19.9	20.0	21.9	0.412	0.0670
MEI (MJ/d)	16.9	16.9	18.9	0.417	*
DE/GE (MJ/MJ)	0.761^a^	0.705^b^	0.750^a^	0.00824	**
ME/GE (MJ/MJ)	0.646	0.599	0.649	0.00982	*
ME/DE (MJ/MJ)	0.847	0.847	0.864	0.00489	0.167

Apparent Total Tract Digestibility (ATTD)					
DM (kg/kg)	0.736^a^	0.680^b^	0.729^a^	0.00840	**
OM (kg/kg)	0.784^a^	0.732^b^	0.774^a^	0.00740	**
DOMD (kg/kg)	0.711^a^	0.667^b^	0.703^a^	0.00727	**
NDF (kg/kg)	0.776^a^	0.679^b^	0.755^a^	0.0101	**
ADF (kg/kg	0.726^a^	0.600^b^	0.723^a^	0.0130	**
N (kg/kg)	0.708^a^	0.627^b^	0.691^a^	0.00973	**
GE (MJ/MJ)	0.761^a^	0.705^b^	0.750^a^	0.00824	**

SIL, silage; BG, Beagle willow variety; TN, Terra Nova willow variety; DMI, DM intake; OMI, organic matter intake; NDFI, NDF intake; ADFI, ADF intake; CTI, condensed tannin intake; GEI, gross energy intake; DEI, digestible energy intake; MEI, metabolisable energy intake; DE, digestible energy; GE, gross energy; ME, metabolisable energy; DOMD, dry organic matter digestibility; N, nitrogen; The symbol: ‘*’,’**’, ‘***’ denote significance *P* < 0.05, 0.01 and 0.001, respectively. Values within a row with different superscripts differ significantly at *P* < 0.05.

### Condensed tannin-containing treatments had no effects on feed intakes

Concentrate (CDMI), forage (FDMI), organic matter (OMI) and total dry matter intake (TDMI) were not different between forage treatments (*P* > 0.05; Table 2). Regarding fibre, acid detergent fibre intake (ADFI) was different between treatments (*P* < 0.05, Table 2). Treatment TN had a 15% greater ADFI than SIL (*P* < 0.01), whilst all other interactions were non−statistically significant. Differences in neutral detergent fibre intake (NDFI) between treatments were non-significant.

### Treatment Beagle reduced digestible energy available from gross energy intake

**Fig. 3:**
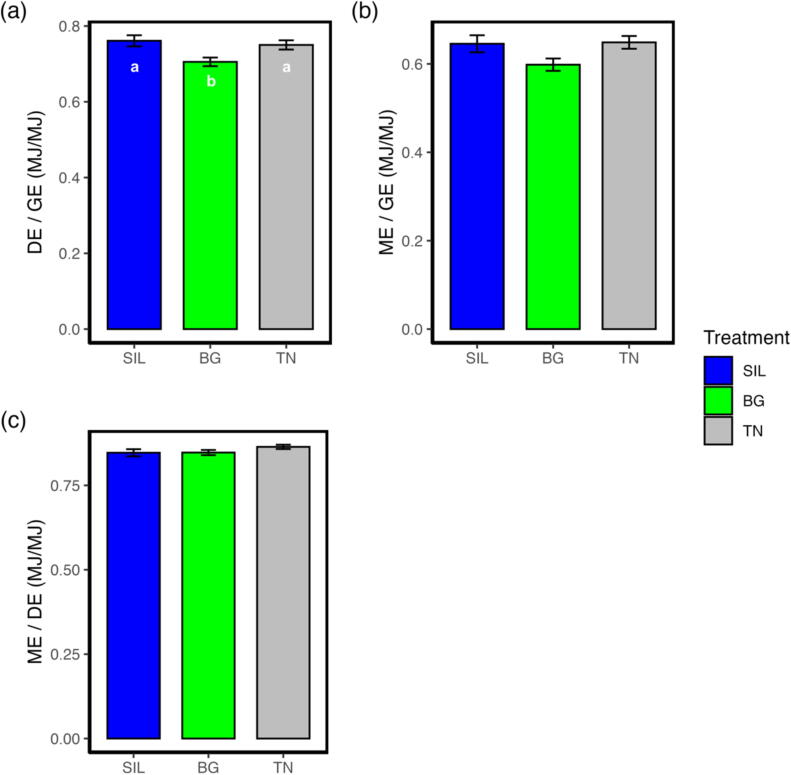
Bar plot showing energy use efficiency of yearling ewes fed three different treatments of different condensed tannin content. (A) DE / GE is the ratio of digestible energy produced relative to gross energy intake. The different lettering ‘a’ and ‘b’ denotes differing in significance <0.05 between feed treatments. (B) ME / GE is the ratio of metabolisable energy produced relative to gross energy intake. C) ME / DE is the ratio of metabolisable energy to digestible energy. SIL, silage; BG, Beagle willow variety; TN, Terra Nova willow variety.

Gross energy intake (GEI) was different (*P* < 0.05; Table 2) by treatment with BG and TN, having an 8 and 10% greater GEI than SIL, respectively (*P* < 0.05). While digestible energy intake (DEI) was not different between treatments (*P* = 0.0670; Table 2), metabolisable energy intake (MEI) differed (*P* < 0.05; Table 2); however, the interactions between treatments were non−statistically significant. The ratio of DEI to GEI (DE/GE; Fig. 3a) was different by treatment (*P* < 0.01; Table 2), with BG being 8 and 6% lower than SIL and TN, respectively (*P* < 0.05). No differences in DE/GE were observed between TN and SIL (*P* = 0.550). Comparably, the ratio of MEI and GEI (ME/GE; Fig. 3b) was different by treatment (*P* < 0.05; Table 2); however, interactions between treatments were non−statistically significant. The ratio of MEI and DEI (ME/DE; Fig. 3c) was not different between treatments (*P* = 0.167; Table 2).

### Apparent total tract digestibility was reduced for Beagle treatment

**Fig. 4:**
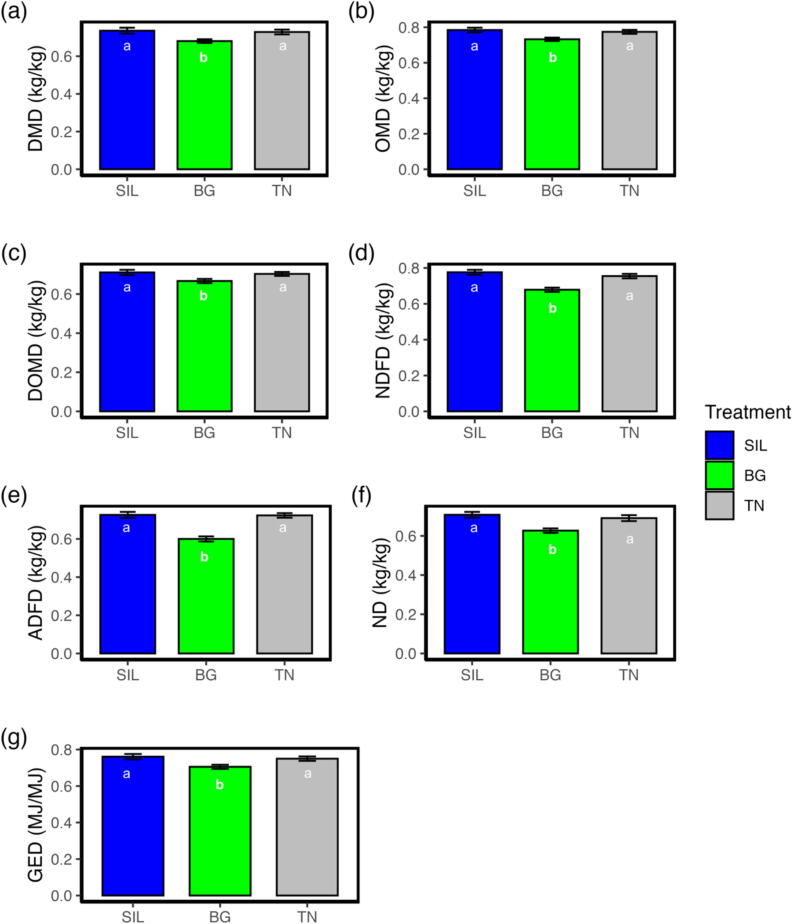
Bar plot showing the apparent total tract digestibility of yearling ewes fed three different treatment diets of differing condensed tannin content. (A) DMD is the DM digestibility. (B) OMD is the organic matter digestibility. (C) DOMD is the digestible organic matter in the DM. (D) NDFD is the NDF digestibility. (E) ADFD is the ADF digestibility. (F) ND is the nitrogen digestibility. (G) GED is the gross energy digestibility. The different lettering ‘a’ and ‘b’ denotes differing in significance <0.05 between feed treatments. SIL, silage; BG, Beagle willow variety; TN, Terra Nova willow variety.

All aspects of digestibility were different among treatments, with BG most responsible for reduced digestibility. DM (Fig. 4a) digestibility for BG had an 8 and 7% reduction relative to SIL and TN, respectively (*P* < 0.05; Table 2) while no differences were observed between TN and SIL (*P* = 0.706). Organic matter (OM; Fig. 4b) digestibility for BG was 7 and 6% lower than SIL (*P* < 0.01) and TN (*P* < 0.05), respectively, with no differences between TN and SIL (*P* = 0.528; Table 2). The digestible organic matter in dry matter (DOMD; Fig. 4c) in BG was 7% lower than SIL (*P* < 0.05), while no differences occurred with TN (*P* = 0.052) and between TN and SIL (*P* = 0.645; Table 2).

Regarding fibre, NDF digestibility (Fig. 4d) was 14 and 11% lower for BG relative to SIL (*P* < 0.001; Table 2) and TN (*P* < 0.001), respectively. Similarly, ADF digestibility (Fig. 4e) was reduced by 21% for BG relative to SIL and TN (*P* < 0.001; Table 2). Digestibility of N (Fig. 4f) for BG was 13 and 10% lower than SIL (*P* < 0.001) and TN (*P* < 0.01), respectively. Lastly, GE digestibility (Fig. 4g) of BG was 8 and 6% lower relative to SIL and TN, respectively (*P* < 0.05).

### Treatment Beagle caused a shift in nitrogen excretion from urine to faeces

Nitrogen outputs in terms of faecal N (FN) were different for all treatments (*P* < 0.001; Table 3). Treatment BG was 32 and 13% greater than SIL (*P* < 0.001) and TN (*P* < 0.05), respectively, while TN was 22% greater than SIL (*P* < 0.01). Conversely, urine N (UN; Table 3) did not differ among treatments (BG = 14.859; SIL = 14.713; TN = 15.683; *P* = 0.542). Total manure nitrogen (MN) output differed by treatment and was greater in both BG and TN compared to silage (*P* < 0.05; Table 3); however, interactions between treatments were non−statistically significant. Overall, absorbed N (AN) output differed by treatment (*P* < 0.001; Table 3) with TN having a 14 and 15% greater AN relative to BG (*P* < 0.05) and SIL (*P* < 0.05); no differences were observed between BG and SIL (*P* = 0.796). Subsequently, retained N (RN) was 26 and 27% greater in TN than BG (*P* < 0.05) and SIL (*P* < 0.05), respectively, while no differences occurred between BG and SIL (*P* = 0.796).

**Table 3:** Nitrogen use efficiency of yearling ewes fed different feed treatments with differing condensed tannin inclusion.

	Treatment				
Item	SIL	BG	TN	SEM	*P*-value
Nitrogen intake and output (g/d)					
NI	33.2^a^	38.0^b^	40.2^b^	0.802	**
FN	9.67^a^	14.2^b^	12.4^c^	0.462	***
UN	14.7	14.9	15.7	0.715	0.542
MN	24.4	29.1	28.1	1.00	*
AN	23.5^a^	23.9^a^	27.8^b^	0.642	***
RN	8.78^a^	8.99^a^	12.1^b^	0.698	*

Nitrogen utilisation efficiency (g/g)					
FN/NI	0.292^a^	0.373^b^	0.309^a^	0.0973	**
UN/NI	0.442	0.385	0.386	0.0163	**
MN/NI	0.734	0.758	0.695	0.0194	0.444
AN/NI	0.708^a^	0.627^b^	0.699^a^	0.00973	**
RN/NI	0.266	0.242	0.305	0.0194	0.444
UN/MN	0.596^a^	0.500^b^	0.554^ab^	0.0129	***
FN/MN	0.404^a^	0.500^b^	0.446^ab^	0.019	***
UN/FN	1.55^a^	1.04^b^	1.27^ab^	0.0655	***
RN/AN	0.369	0.385	0.434	0.0260	0.299

SIL, silage; BG, Beagle willow variety; TN, Terra Nova willow variety; NI, nitrogen intake; FN, faecal nitrogen; UN, urinary nitrogen; MN, manure nitrogen; AN, absorbed nitrogen; RN, retained nitrogen; The symbol: ‘*’,’**’, ‘***’, denote significance *P* < 0.05, 0.01 and 0.001, respectively. Values within a row with different superscripts differ significantly at *P* < 0.05.

**Fig. 5:**
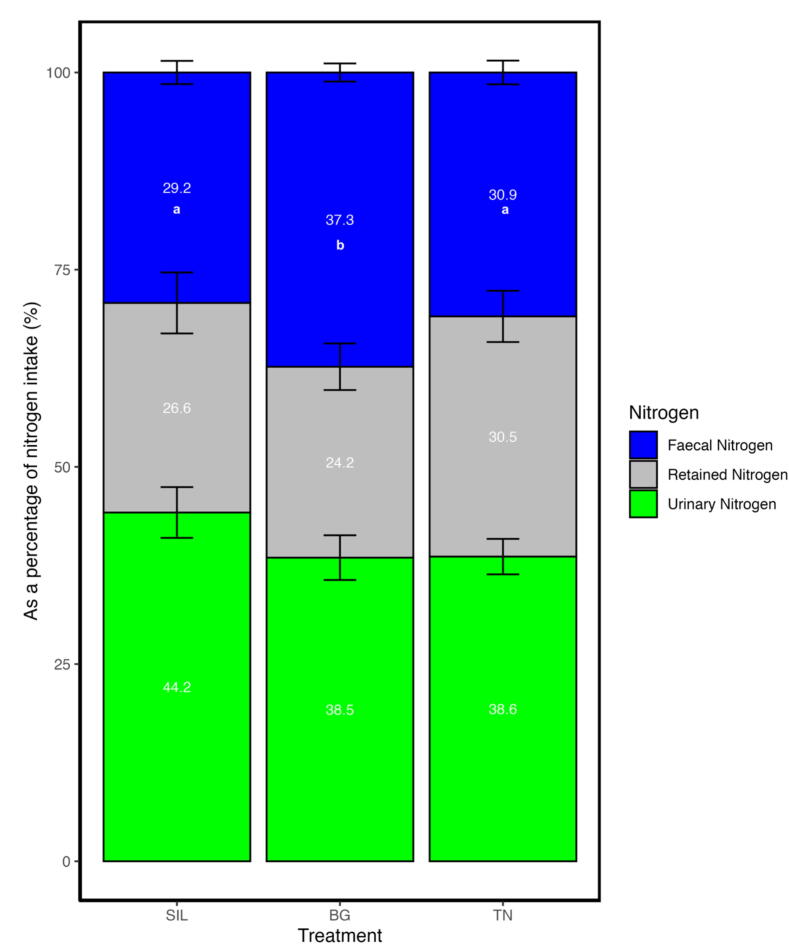
Stacked bar plot showing nitrogen apportioning as a proportion of nitrogen input of yearling ewes fed three different treatment diets of differing condensed tannin content. The blue colour represents the proportion of nitrogen apportioned to faecal excretion as a proportion of nitrogen input. The grey colour represents the proportion of nitrogen retained for growth and repair as a proportion of nitrogen input. The green colour represents the proportion of nitrogen apportioned to urine excretion as a proportion of nitrogen input. The different lettering ‘a’ and ‘b’ denotes differing in significance <0.05 between feed treatments. SIL, silage; BG, Beagle willow variety; TN, Terra Nova willow variety.

**Fig. 6:**
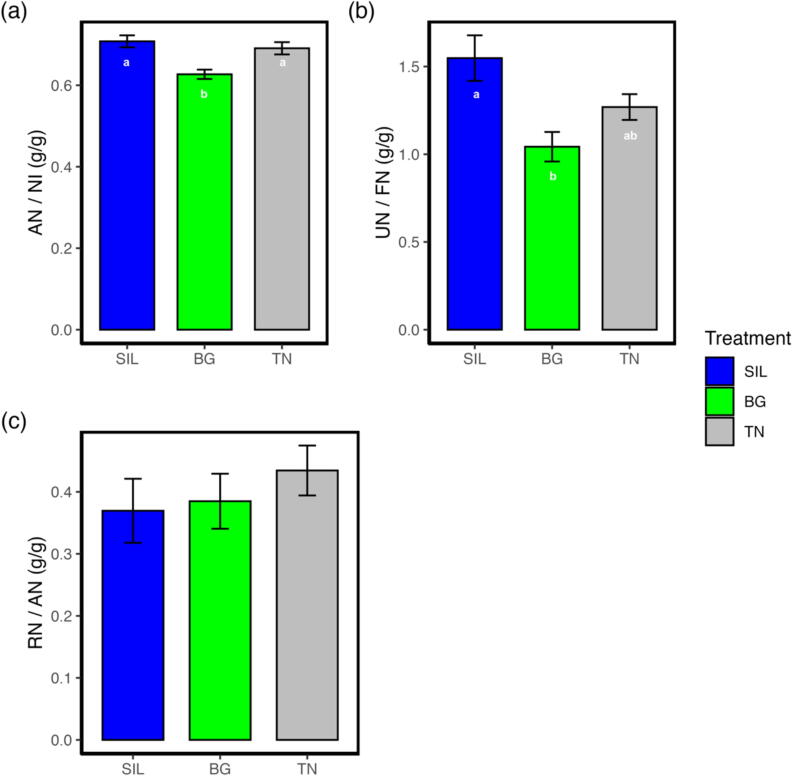
Bar plot showing nitrogen use efficiency of different parameters of yearling ewes fed three different treatment diets of differing condensed tannin content. (A) AN / NI shows the ratio of absorbed nitrogen as a proportion of nitrogen intake. (B) UN / FN shows the ratio of urinary nitrogen as a proportion of faecal nitrogen content. (C) RN / AN shows the ratio of retained nitrogen of the yearling ewe as a proportion of the absorbed nitrogen. The different lettering ‘a’ and ‘b’ denotes differing significance <0.05 between feed treatments. SIL, silage; BG, Beagle willow variety; TN, Terra Nova willow variety.

In terms of utilisation, the proportion of FN to NI (FN/NI; Fig. 5) was affected by treatment (*P* < 0.01; Table 3), where BG had 22 and 17% greater FN/NI relative to SIL (*P* < 0.001) and TN (*P* < 0.01). The proportion of UN to NI (UN/NI; Fig. 5) was also affected by treatment (*P* < 0.01) and tended to be lower in BG and TN relative to SIL, despite interactions between treatments non−statistically significant. No treatment effect (*P* = 0.444; Table 3) was observed for the proportion of RN to NI (RN/NI; Fig. 5). Total MN as a proportion of NI (MN/NI) was not different between treatment (*P* = 0.444; Table 3). The AN as a proportion of NI (AN/NI; Fig. 6a) was affected by treatment (*P* < 0.01); ewes consuming BG showed a 13 and 12% lower AN/NI relative to SIL (*P* < 0.001) and TN (*P* < 0.01), respectively.

The proportion of UN to MN (UN/MN; Table 3) was affected by treatments (*P* < 0.001). Treatment BG had a 16% lower proportion of UN/MN relative to SIL (*P* < 0.01, while the proportion of FN to MN (FN/MN) was 19% higher in BG relative to SIL (*P* < 0.01). Moreover, the UN to FN proportion (UN/FN; Fig. 6b) was 48% lower (*P* < 0.01) for BG compared to the SIL. Finally, the proportion of RN to AN (RN/AN; Fig. 6c) was similar among all the treatments (*P* = 0.299; Table 3).

### Metabolites showed some differences among feed treatments

No significant differences were observed between NMR buckets of blood and urine metabolites from different feed treatments. Nevertheless, 26 blood and 22 urine metabolites were selected and extracted from the Chemonx spectral software analysis based on specific metabolic pathways with the potential to be affected by CT inclusion such as protein and carbohydrate.

**Fig. 7:**
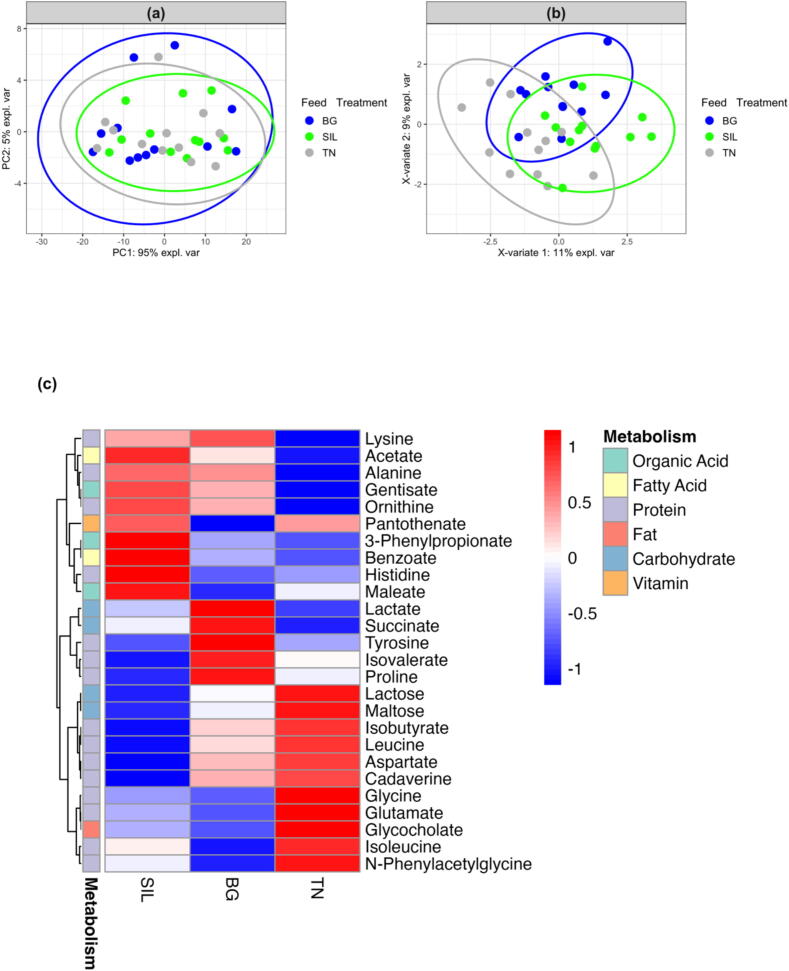
Exploratory analysis of blood metabolites from yearling ewes fed three different feed treatments of differing condensed tannin content. (A) Principal Component Analysis plot of blood metabolites grouped according to feed treatment. PC1 and PC2 account for 95 and 5% respectively of the total variation. (B) Partial Least Squares Discriminant Analysis plot of blood metabolites grouped according to feed treatment. X-variate 1 and X-variate 2 account for 11 and 9% respectively of the variation. (C) Heatmap of different blood metabolite concentrations classified according to the main type of metabolism they are involved in for the three different feed treatments of differing condensed tannin content. Hierarchical clustering analysis of the average metabolite concentration was calculated using Euclidean distance resulting in a scale of +1 (Red: higher concentration) to −1 (Blue: lower concentration). SIL, silage; BG, Beagle willow variety; TN, Terra Nova willow variety.

**Fig. 8:**
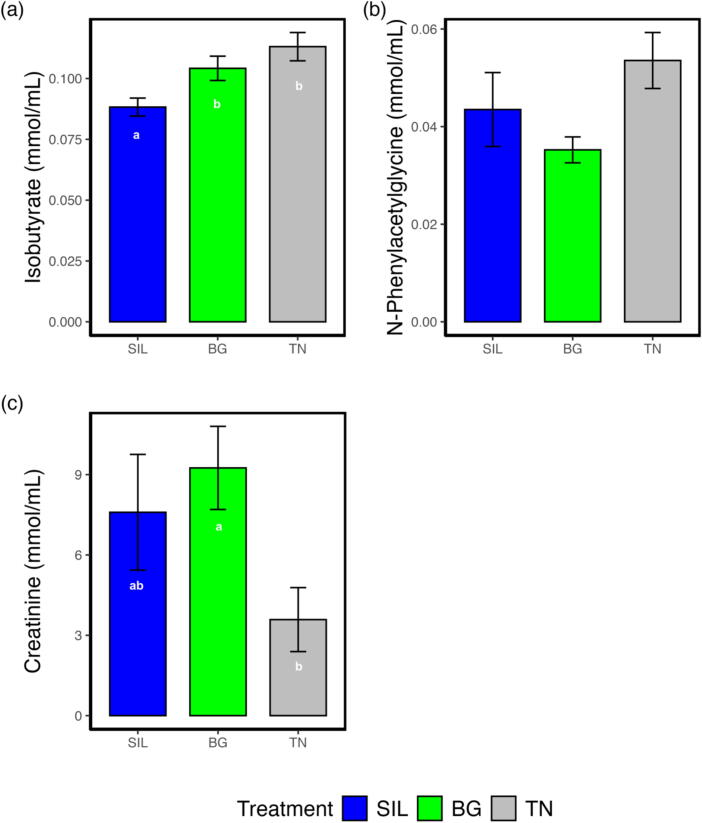
Bar plot of significantly different blood and urine metabolite concentrations (mmol/mL) in ewes because of feed treatment. (A) Isobutyrate is a blood metabolite involved in protein metabolism. (B) N-Phenylacetylglycine is a blood metabolite involved in protein metabolism. (C) Creatinine is a urine metabolite involved in protein metabolism. The different lettering ‘a’ and ‘b’ denotes differing in significance <0.05 between feed treatments. SIL, silage; BG, Beagle willow variety; TN, Terra Nova willow variety.

Fig. 7 displays the exploratory analysis of blood metabolites with principal component analysis (Fig. 7a) showing no clear separation by feed treatment with PC1 and PC2 accounting for 95 and 5% respectively of the total variation. However, some degree of separation between feed treatments was observed using the PLS-DA supervised model for predictive and descriptive modelling albeit with high variability (Fig. 7b). Fig. 7c visualises the relationships and differences in the concentrations of blood metabolites among feed treatments using hierarchical cluster analysis. Metabolites associated with protein digestion exhibited the highest concentrations in TN, followed by intermediate levels in BG, and the lowest concentrations in SIL. The same was also observed in metabolites associated with carbohydrate digestion. Metabolites associated with organic acid, fatty acid and vitamin digestion had higher concentrations in SIL compared to BG and TN. Univariate analysis of blood showed that some compounds involved in protein metabolism, isobutyrate (Fig. 8a) and N-phenylacetylglycine (Fig. 8b), were significantly affected by feed treatment. Isobutyrate concentration was 17 and 28% higher in BG and TN, respectively, relative to SIL (*P* < 0.01; Supplementary Table S4, row 8), while concentration of N-phenylacetylglycine was affected by feed treatment (*P* < 0.05; Supplementary Table S4, row 13) and tended to be lowest for BG relative to SIL and TN although interactions between feed treatments were non−statistically significant.

**Fig. 9:**
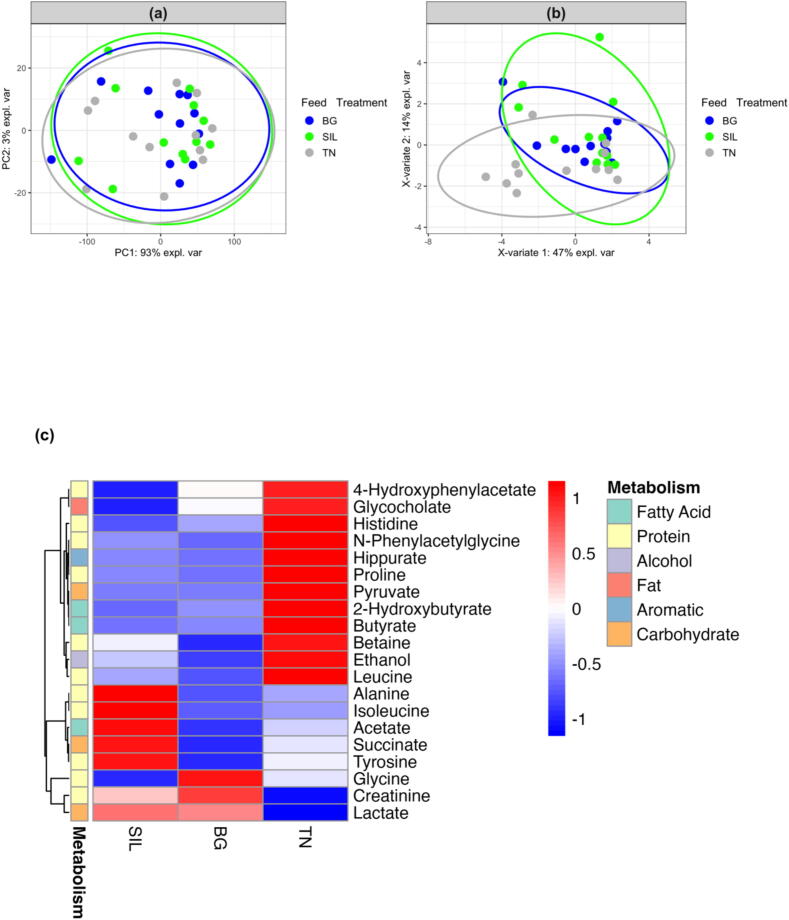
Exploratory analysis of urine metabolites from yearling ewes fed three different feed treatments of differing condensed tannin content. (A) Principal Component Analysis plot of urine metabolites grouped according to feed treatment. PC1 and PC2 account for 93 and 3% respectively of the total variation. (B) Partial Least Squares Discriminant Analysis plot of urine metabolites grouped according to feed treatment. X-variate 1 and X-variate 2 account for 47 and 14% respectively of the variation. (C) Heatmap of different urine metabolite concentrations classified according to the main type of metabolism they are involved in for the three different feed treatments of differing condensed tannin content. Hierarchical clustering analysis of the average metabolite concentration was calculated using Euclidean distance, resulting in a scale of +1 (Red: higher concentration) to −1 (Blue: lower concentration). SIL, silage; BG, Beagle willow variety; TN, Terra Nova willow variety.

The same approach was followed for the analysis of urine metabolites (Fig. 9). Principal component analysis (Fig. 9a) shows no separation according to feed treatment, with PC1 and PC2 responsible for 93 and 3% of the variation. While the PLS-DA supervised model (Fig. 9b) showed some variation between feed treatments again with high variability. Hierarchical cluster analysis of urine metabolites (Fig. 9c) showed that most urine metabolites associated with protein digestion were highest in TN. However, alanine, isoleucine and tyrosine concentrations were highest in SIL. Alternatively, glycine and creatinine concentrations were highest in BG. Carbohydrate-classified metabolites showed a similar variation, with the concentration of pyruvate highest in TN, succinate highest in SIL and lactate highest in both SIL and BG. Aromatic metabolites were highest in TN. The concentration of fat metabolites in urine was highest in TN and decreased by the order of BG and lowest in SIL. Fatty acid metabolite concentrations were highest in TN while alcohol metabolites were highest in TN and reduced in concentration in the order SIL followed by BG. Univariate analysis of urine metabolites showed a 61% lower (*P* < 0.05; Supplementary Table S5, row 4) concentration of creatinine (Fig. 9c) in TN relative to BG.

Therefore, with hierarchical cluster analysis showing differences and moderate separation in PLS-DA between blood and urine metabolites, feed treatment (CTI) could be influencing metabolism. However, variability was high and further studies using larger sample sizes would be needed to validate these findings.

## Discussion

Despite the potential of willow fodder in animal nutrition, this is the first pioneering study to investigate its digestibility through *in vivo* animal trials and assess the role and impact of its condensed tannins on the metabolic pathways related to digestion.

### Energy utilisation and efficiency

The rate and extent of fermentation of dietary carbohydrates in the rumen are the main drivers behind the energy supply for the animal (Hall, 2004). Therefore, efficient utilisation of this energy is vital as it determines productivity. Low digestible structural carbohydrates reduce digestible energy (DE) availability and subsequently, less metabolisable energy (ME) is available for maintenance energy requirements, restricting productive responses (Steinfeld et al., 2006, Beauchemin et al., 2009, Gerber et al., 2013).

Among the treatments, the two CT-containing ones had a higher GE content compared to the SIL. On the other hand, the BG (higher CTI) had a significantly lower DE yield as a proportion of GE intake compared to SIL and TN. These changes in energy intake and digestibility suggest that willow as a feed may have contributed to alterations in nitrogen partitioning independent of CT effects. This indicates that the energy utilisation of BG may have been influenced by its CTI of 1.20% DM, as CTs can inhibit digestibility. Similarly, in a study where cattle were fed diets with 0, 1, 2, and 3% CT/Kg DM, no significant differences in DE/GE were observed (0%: 0.55; 1%: 0.53; 2%: 0.51; 3%: 0.48) (Piñeiro-Vázquez et al., 2017). However, by increasing the CT content to 4%, a significant reduction (*P* < 0.05) in DE/GE occurred, resulting in decreases of 22, 19, 16, and 10% relative to the lower CT inclusions.

The ratio of ME/GE was significantly different (*P* < 0.05) among treatments and, despite no significant interaction between feed treatments, was numerically lower for BG. Nevertheless, no differences occurred for methane energy (CH_4_-E) or urine energy losses among the treatments, indicating that a CTI of 1.20% did not significantly enhance metabolisable energy yield, nor strongly inhibit methanogenesis. This reduction was due to a decline in digestible energy availability, as confirmed by a similar ME/DE ratio across the treatments. Conversely, previous studies demonstrated that CTs can reduce energy loss to methane, increase ME availability, and improve energy use efficiency. When goats were fed Lespedeza cuneata with a CTI of 20% DM, CH_4_ production decreased by 27% alongside a 7% increase in ME availability (0.5 MJ/d) (Puchala et al., 2012). However, most studies report a decline in CH_4_ energy because higher CTI is associated with reductions in ME availability. By incrementing CTI from 2, 3, and 4%, energy loss as CH_4_ reduced linearly by 31, 48, and 57%, respectively, compared to the 0% CTI treatment when cattle were supplemented with quebracho CT extract (Piñeiro-Vázquez et al., 2017). However, at 4% CTI, the concentration of ME availability was reduced by 29% relative to the control, while no differences were observed between 1, 2, or 3% CTI treatments. Likewise, dairy cows offered a diet with 1.46% CTI from *Acacia* mearnsii showed a 22.3% decrease in CH_4_ emissions and a 14.3% reduction in gross energy utilisation efficiency (Grainger et al., 2009). Reductions in ME availability as CTI increased result from higher energy excretion in faeces due to declines in rumen degradability, DM, and OM digestibility, which is partly due to the formation of complexes between CT and protein or carbohydrates (McSweeney et al., 2001).

### Apparent total tract digestibility

Favourable effects of CTs on ruminant nutrition involve CTs reducing nutrient degradation in the rumen through forming complexes. An important interaction is particularly with protein, followed by CT releasing nutrients in the lower digestive tract (lower pH) which can increase nutrient absorption and potentially improve digestibility aiming to increase nutrient absorption and improve digestibility (Frutos et al., 2004). However, reduced digestible energy availability for BG (1.20% CTI) in the current study is likely due to a reduced digestibility across all nutrients relative to SIL and TN (0 and 0.07% CTI, respectively), indicating that higher CTI may negatively affect digestibility. BG resulted in significant reductions in DM, OM and DOMD relative to SIL and TN. Therefore, microbial digestion and fermentation efficiency appeared impaired with BG.

It has been confirmed that protozoa are responsible for 50% of the fibrolytic activity in the rumen (Coleman, 1986), suggesting that fibrolytic bacteria and protozoa may have been specifically affected by the higher CTI. In a similar study, the digestibility of OM was unaffected at CT1 (1% CT inclusion) but strongly decreased by 21% for CT3 (3% CT inclusion) and 28% for CT5 (5% CT inclusion) (Gerlach et al., 2018). With the same CT source (*Acacia mearnsii*) and inclusion rate of 2.5%, OM digestibility was reduced by 3% in a beef trial (Koenig and Beauchemin, 2018). Conversely, with quebracho CT, a 3% CTI did not affect OM digestibility (Dschaak et al., 2011). Therefore, the impact of CT on DM and OM digestibility appears to depend on the CT source and inclusion level. However, the inclusion of BG CTs appears to fall within the range associated with a reduction in OM digestibility. A reduction in OM digestibility led to decreased NDF and ADF digestibility for BG compared to TN and SIL, highlighting that the higher CT inclusion can negatively affect fibre digestion. This is an indication that the different fibre fractions were bound to CT. This finding is supported by other studies, such as Henke et al. (2017), which observed a reduction in OM digestibility, with an even greater impact on fibre fractions, in dairy cows fed a quebracho CT extract. Using the same CT source, another study did not find a reduction in NDF digestibility at 0.4% (Mezzomo et al., 2011) or 0.6% (Baah et al., 2007) inclusion rates.

Affinity of CT with N is the greatest compared to other macronutrients (Mezzomo et al., 2011), and consequently, BG had a 13 and 10% decrease in N digestibility relative to SIL and TN, respectively. These results suggest that for BG, dissociation of the CT-N complex may not have occurred with the pH drop in the abomasum, resulting in no increase in N uptake by the animal (Aerts et al., 1999). This same effect had been observed in quebracho CT, where digestibility of CP decreased by 5 and 15% with a 1 and 2% CT inclusion rate, respectively (Beauchemin et al., 2007). Moreover, when CT containing *Lotus corniculatus* (22 g CT/kg DM) was fed as the sole diet, N digestibility was reduced by 7–8% (Waghorn et al., 1987), whereas *Lotus pedunculatus* had a lesser effect on CP digestibility (Waghorn and Shelton, 1997). However, it was determined that CP digestibility was not affected with a CT inclusion rate of 0.8% from *Acacia and Leucaena* (Soltan et al., 2013). Thus, the effect of CT on N digestibility depends on CT:N ratio, which is influenced by molecular weight, source, structure and the amino acid profile of the proteins (Min et al., 2003).

CTs with greater mDP content are more potent with protein precipitation while greater PD content results in more sites for hydrogen bonding to occur (Jones et al., 1976, Naumann et al., 2018). Therefore, these structural characteristics can influence the ability of CTs to form complexes with protein and fibre. In this study, BG CTs had an mDP of 7.3 and PD content of 62.70% which likely contributed to the observed reductions in nitrogen and fibre digestibility at a low CT inclusion of 1.2% DM. Conversely, TN CTs had a much greater PC content (94.57%) compared to BG. However, for TN, the CT inclusion level of 0.07% of TDMI was probably too low to exert measurable effects on protein and fibre binding, and consequently on digestibility, energy, and NUE.

In addition to CT binding, the contribution of willow as a feed should also be considered. Both BG and TN had significantly higher nitrogen intake than SIL, as observed in the present study, reflecting the contribution of willow to dietary N supply. This increase in dietary N supply likely contributed to shifts in N excretion patterns, independent of CT effects, suggesting that the nutritional composition of willow must be accounted for alongside its bioactive compounds when interpreting nitrogen utilisation outcomes.

In this study, it was observed that GE digestibility of BG was reduced compared to TN and SIL. While CTs can bind structurally to N and fibre, they may also influence rumen microbiome function. It has been suggested that CT can prevent or interfere with the attachments of rumen microbes to feed particles (Frutos et al., 2004). They are also chelating agents, reducing the availability of certain metallic ions necessary for microbial metabolism and can inhibit enzymes, thereby affecting microbial fermentation pathways (McSweeney et al., 2001). These combined effects may help explain the GE digestibility reductions observed in our study. At the same inclusion rate used for BG in our study, a similar study with *Acacia mearnsii* reported a 3.2% reduction in GE digestibility (Koenig and Beauchemin, 2018).

### Nitrogen utilisation and efficiency

The effect of CTs on nitrogen balance remains unclear and inconsistent across the literature (Jayanegara et al., 2012). The two willow treatments (BG and TN) used in this study resulted in a higher NI due to their higher N content compared to SIL. Nevertheless, the reduced AN/NI for BG, compared to SIL and TN, suggests that the CTs may have been present at a level sufficient to bind nitrogen in the rumen, forming complexes, and reducing degradability by the rumen microbiome (Beauchemin et al., 2007). In normal cases, rumen-degradable protein and non-protein nitrogen are degraded by rumen microorganisms to ammonia (NH_3_), amino acids and peptides. While NH_3_ is the primary product and major N source for ruminal microbial protein synthesis, it accounts for 20–35% of lost dietary N in the rumen (Mueller-Harvey, 2006).

Accordingly, the UN/NI was significantly affected by treatment and tended to be lower for BG and TN compared to SIL. This result suggests that for BG, a smaller proportion of ruminal NH_3_ may have been incorporated into bacterial protein or absorbed across the rumen epithelium, resulting in less N being transported to the liver and excreted in urine relative to N intake. A similar result was observed in a study where a diet consisting of 40% distiller’s grain, with or without CT (2.5% CTI), resulted in a 17% decrease in the UN/NI ratio for the CT treatment (Koenig and Beauchemin, 2018).

Despite the impact CTs had in the rumen, RN/NI was non-significant among all the treatments. This result indicated that in the abomasum or small intestine, CTs in BG may not have dissociated from protein despite the drop in pH (Molan et al., 2001). While the impact of CT on intestinal function is not well understood, it has been suggested that in the more neutral pH of the small intestine, CT may become reactivated, potentially rebinding to proteins and reducing nutrient uptake and nitrogen retention (Waghorn, 2008). Furthermore, CTs may complex with intestinal enzymes and mucosa which could further disrupt nitrogen uptake (Kariuki and Norton, 2008). This output was also found in other studies (Tiemann et al., 2008, Aghamohamadi et al., 2014) where no changes were observed in nitrogen retention, though faecal N increased with a lower N excretion in urine. Additionally, a previous study concluded that increasing the CTI level from 0.86 to 1.4% caused an increase in the level of N excretion in the faeces, while RN and UN decreased (Grainger et al., 2009). Conversely, a study shows CTs can increase RN (Min et al., 2001). Condensed tannins in both *Lotus corniculatus* and *Lotus pedunculatus* increased the abomasal/duodenal non-ammoniacal nitrogen flow relative to control; however, only CTs in *Lotus corniculatus* significantly increased the absorption of essential amino acids from the small intestine. The differing effects between the two species may be due to structural differences in the CTs, as CTs from *Lotus corniculatus* did not affect the apparent digestibility (% abomasal flow) of essential amino acid in the small intestine, whereas CTs in *Lotus pedunculatus* markedly reduced apparent N digestibility in the small intestine (Min et al., 2001). Similar results were found in another study, where differences in digestion and absorption of amino acid by CT action were suggested to be associated with differences in structural chemistry (Bermingham et al., 2001).

With no changes in MN/NI between treatments, the BG treatment with 1.20% CTI did not increase overall nitrogen utilisation or efficiency but instead resulted in an increase in FN/NI. This indicates that a shift in nitrogen deposition occurred with BG, from urine to faeces, which could help minimise NH3 volatilisation to the environment (Gill et al., 2010). Similar results were observed when increasing proportions of CT-rich legumes in the diet produced linear increases in faecal N losses relative to N intake, while urinary N losses decreased (Tiemann et al., 2008). However, unlike the present study, levels of retained N were positive for CT-containing diets, except for the high CT-containing Calliandra diet relative to control. These findings suggest that the addition of CT does not always enhance nitrogen retention (Carulla et al., 2005). Moreover, it has been suggested that ruminants may benefit from CT supplementation when the increase in duodenal protein flow exceeds any decrease in amino acid absorption in the intestine.

### Impact of condensed tannins on metabolites

Consistent with our study, Costa et al. (2021) found no effects on blood metabolites when supplementing *Acacia mearnsii* at CTI levels of 0, 2, 4, 6, and 8% DM. However, they observed a reduction in average daily gain (*P* < 0.001) in lambs with a CTI greater than 2%. Nevertheless, upon visualising the heatmap, it is evident that the average concentration of metabolites associated with protein digestion is lower in BG compared to TN, despite both having the same NI. On the other side, protein metabolites are still lower in SIL, and this is due to the significantly lower NI in this diet compared to the CT treatments.

The metabolites associated with carbohydrate digestion were found to be lower in BG compared to TN, which aligns with the results showing that BG led to reductions in energy utilisation, digestibility, and AN. This result suggests that at a CTI level of 1.20% DM, the BG CTs were included at too high a level, preventing dissociation in the abomasum and limiting digestibility and nutrient uptake into the blood. Alternatively, the structure of BG CT resulted in a strong bonding with protein and fibre, also limiting dissociation in the abomasum. For TN, the significant increase in AN into the blood was reflected well in the heatmap and the significant change in compounds involved in protein metabolism (isobutyrate and N-phenylacetylglycine), of which the concentrations were the highest in TN. With a lower CTI than BG, less protein was bound in the rumen, allowing for more absorption. This suggests that the TN CTs may have dissociated in the abomasum.

Regarding urine metabolites, BG had the lowest concentration of metabolites associated with protein digestion. The BG diet, with the higher CTI, resulted in a shift in nitrogen deposition, increasing nitrogen excretion, which was apportioned to faecal nitrogen. At the lower CTI level, TN (0.07% DM) had the highest concentration of protein metabolites in the urine. While TN had the greatest AN, there was no difference in RN between treatments, resulting in the excess nitrogen being excreted in urine. A similar study involving quebracho tannin extract at different inclusion levels (0, 1, 2, 4, 6% DM) measured the impact on apparent nutrient digestibility, nitrogen balance and urinary purine derivatives excretion in heifers (Ahnert et al., 2015). In agreement with our study, it was found an increase in CTI resulted in both a significant linear decrease in urinary nitrogen excretion and a linear increase in faecal nitrogen excretion. Urinary purine derivative excretion declined from 103 mmol/d to 80 mmol/d when CTI increased from 1 to 6%, indicating a 36% decrease in estimated duodenal microbial CP flow (Ahnert et al., 2015). However, this study concluded that the increase in rumen-escape protein of diets supplemented with CT will not always lead to greater postruminal amino acid absorption and improvements in cattle performance at moderate to high CTI due to pronounced reductions in estimated duodenal MCP flow and an increase in faecal N excretions.

## Conclusion

This pioneering study explored willow’s potential contribution in animal nutrition and sustainable agriculture. The content and structure of CTs varied among willow varieties and influenced nutrient binding capacity and digestibility. In our study, dietary inclusion of Salix ‘Beagle’ reduced the digestibility of dry and organic matter, fibre, nitrogen, and gross energy. CTs’ impact on digestion and feed nutritive value depends on their content and structure and these characteristics need to be taken into consideration when formulating diets. On the other hand, the inclusion of the Beagle variety in the diet of ruminants at 21% DM resulted in a shift of nitrogen excretion from urine to faeces. Nitrogen excretion in faeces is a more environmentally stable form and is less likely to volatilise as ammonia, reducing the environmental impact of ruminant livestock production. Willow could offer a promising strategy to reduce ammonia emissions and mitigate the environmental footprint of the ruminant industry.

## Supplementary material

Supplementary Material for this article (https://doi.org/10.1016/j.animal.2025.101698) can be found at the foot of the online page, in the Appendix section.

## Ethics approval

All animal management and experimental procedures were conducted in accordance with the experimental licence granted by the Department of Health, Social Services and Public Safety for Northern Ireland in accordance with the Animal (Scientific Procedures) Act 1986 (Home Office, 1986).

## Data and model availability statement

The data/models were not deposited in an official repository, and the data/models that support the study findings are available from the authors upon request.

## Declaration of generative AI and AI-assisted technologies in the writing process

During the preparation of this work the author(s) did not use any AI and AI-assisted technologies.

## Author ORCIDs

**J. P. Thompson:** https://orcid.org/0000-0002-8188-6258.

**O. Cristobal-Carballo:** https://orcid.org/0000-0001-7880-7590.

**T. Yan:** https://orcid.org/0000-0002-1994-5202.

**W.E. Zeller:** https://orcid.org/0000-0002-1883-4519.

**S. Huws:** https://orcid.org/0000-0002-9284-2453.

**L. Safo:** https://orcid.org/0000-0002-5953-9188.

**A.D. Southam:** https://orcid.org/0000-0003-3030-7663.

**C. Ludwig:** https://orcid.org/0000-0001-8901-6970.

**G.R.Lloyd:** https://orcid.org/0000-0001-7989-6695.

**S. Stergiadis:** https://orcid.org/0000-0002-7293-182X.

**K. Theodoridou:** https://orcid.org/0000-0002-2848-5594.

## CRediT authorship contribution statement

**J.P. Thompson:** Writing – review & editing, Writing – original draft, Visualisation, Validation, Software, Methodology, Investigation, Formal analysis, Data curation, Conceptualisation. **O. Cristobal-Carballo:** Writing – review & editing, Data curation. **T. Yan:** Writing – review & editing, Data curation. **W.E. Zeller:** Writing – review & editing, Methodology, Data curation. **S. Huws:** Writing – review & editing, Supervision. **L. Safoi:** Methodology, Data curation. **A.D. Southam:** Writing – review & editing, Methodology, Data curation. **C. Ludwig:** Writing – review & editing, Methodology, Data curation. **G.R. Lloyd:** Writing – review & editing, Methodology, Data curation. **S. Stergiadis:** Writing – review & editing, Supervision. **K. Theodoridou:** Writing – review & editing, Supervision, Project administration, Methodology, Funding acquisition, Conceptualisation.

## Declaration of interest

The authors declare they have no competing interests.
