# Supplementary material for: Supplementation with willow containing condensed tannins shifted nitrogen excretion from urine to faeces in yearling ewes
Source: Animal. 2025 Dec;19(12):101698. doi: 10.1016/j.animal.2025.101698 (PMC12708440; doi:10.1016/j.animal.2025.101698)
Supplement: Supplementary Data 1 [file mmc1.pdf]

Supplementary material for

Supplementation with willow condensed tannins shifted nitrogen excretion from urine to faeces in yearling ewes

J.P. Thompson, O. Cristobal-Carballo, T. Yan, W.E. Zeller, S. Huws, L. Safo, A.D. Southam, C. Ludwig, G.R. Lloyd, S. Stergiadis, K. Theodoridou

***animal* journal**

Table of contents

Supplementary Table S1. Chemical composition of all dietary components used in the study 2

Supplementary Table S2. Metabolic pathways and main metabolic classification of blood and urine metabolites examined in this study 3

Supplementary Table S3. Nutritional composition of the three formulated diets 19

Supplementary Table S4. Blood metabolite concentration (mmol/mL) of yearling ewes fed diets with differing condensed tannin inclusion 20

Supplementary Table S5. Urine metabolite concentration (mmol/mL) of yearling ewes fed diets with differing condensed tannin inclusion 21

Supplementary Table S 1.

Chemical composition of all dietary components used in the study

	Nutritional Composition			
	SIL	BG	TN	C
DM (g/kg fresh)	259.61	326.87	267.73	883.29
Ash (g/kg DM)	89.94	71.56	80.86	96.70
OM (g/kg DM)	910.06	928.44	919.14	903.30
ADF (g/kg DM)	347.87	303.82	391.82	152.53
NDF (g/kg DM)	543.74	385.69	509.01	245.14
Starch (g/kg DM)	-	-	-	258.22
EE (g/kg DM)	43.56	21.21	37.68	4040
N (g/kg DM)	24.77	34.61	37.71	27.92
CP (g/kg DM)	154.83	216.30	235.67	174.48
GE (MJ/kg DM)	19.50	20.16	20.18	17.72
ME (MJ/kg DM)	10.78	10.348	12.44	13.00

SIL, Silage control; BG, Salix. Beagle; TN, Salix Terra Nova; C, Concentrate; EE, ether extract; N, nitrogen; GE, gross energy; ME, metabolisable energy;

Supplementary Table S 2.

Metabolic pathways and main metabolic classification of Blood and Urine metabolites of yearling ewes

Row	Biofluid	Metabolite	Metabolism Pathways	Metabolic Classification
1	Urine	2-Hydroxybutyrate	Propanoate metabolism	Fatty Acid
2	Urine	4-Hydroxyphenylacetate	Tyrosine metabolism<br>Phenylalanine metabolism	Protein
3	Blood	3-Phenylpropionate	Ethylbenzene degradation<br>Microbial metabolism in diverse environments<br>Degradation of aromatic compounds	Organic acids
4	Blood<br>Urine	Acetate	Glycolysis / Gluconeogenesis<br>Taurine and hypotaurine metabolism<br>Phosphonate and phosphinate metabolism<br>Glycosaminoglycan biosynthesis - heparan sulfate / heparin<br>Pyruvate metabolism<br>Glyoxylate and dicarboxylate metabolism<br>C5-Branched dibasic acid metabolism<br>Methane metabolism<br>Other carbon fixation pathways<br>Zeatin biosynthesis<br>Sulfur metabolism<br>Metabolic pathways<br>Biosynthesis of secondary metabolites<br>Microbial metabolism in diverse environments<br>Carbon metabolism<br>Degradation of aromatic compounds<br>Cholinergic synapse<br>Alcoholic liver disease<br>Carbohydrate digestion and absorption<br>Protein digestion and absorption	Fatty Acid
5	Blood<br>Urine	Alanine	Cyanoamino acid	Protein
6	Blood	Aspartate	Arginine biosynthesis	Protein

			Alanine, aspartate and glutamate metabolism<br>Glycine, serine and threonine metabolism<br>Monobactam biosynthesis<br>Cysteine and methionine metabolism<br>Lysine biosynthesis<br>Histidine metabolism<br>beta-Alanine metabolism<br>Cyanoamino acid metabolism<br>D-Amino acid metabolism<br>Carbon fixation by Calvin cycle<br>Nicotinate and nicotinamide metabolism<br>Pantothenate and CoA biosynthesis<br>Aminoacyl-tRNA biosynthesis<br>Biosynthesis of various other secondary metabolites<br>Biosynthesis of various antibiotics<br>Biosynthesis of various plant secondary metabolites<br>Biosynthesis of plant secondary metabolites<br>Biosynthesis of alkaloids derived from ornithine, lysine and nicotinic acid<br>Biosynthesis of plant hormones<br>Metabolic pathways<br>Biosynthesis of secondary metabolites<br>Microbial metabolism in diverse environments<br>Carbon metabolism<br>2-Oxocarboxylic acid metabolism<br>Biosynthesis of amino acids<br>Biosynthesis of cofactors<br>ABC transporters<br>Two-component system	

			Bacterial chemotaxis<br>Neuroactive ligand-receptor interaction<br>Protein digestion and absorption<br>Central carbon metabolism in cancer	
7	Blood	Benzoate	Benzoate degradation<br>Dioxin degradation<br>Toluene degradation<br>Biosynthesis of various alkaloids<br>Biosynthesis of secondary metabolites<br>Degradation of aromatic compounds	Fatty Acid
8	Urine	Betaine	Glycine, serine and threonine metabolism<br>One carbon pool by folate<br>ABC transporters<br>Folate transport and metabolism	Protein
9	Urine	Butyrate	Butanoate metabolism<br>Metabolic pathways<br>Carbohydrate digestion and absorption<br>Protein digestion and absorption	Fatty Acid
10	Blood	Cadaverine	Lysine degradation<br>D-amino acid<br>Glutathione<br>Tropane, piperidine and pyridine alkaloid biosynthesis<br>Biosynthesis of plant secondary metabolites<br>Biosynthesis of alkaloids derived from ornithine, lysine and nicotinic acid<br>Biosynthesis of secondary metabolites<br>Protein digestion and absorption	Protein
11	Urine	Creatinine	Arginine and proline metabolism<br>Metabolic pathways	Protein
12	Urine	Ethanol	Glycolysis/Gluconeogenesis<br>Pyruvate metabolism<br>Metabolic pathways<br>Biosynthesis of secondary metabolites	Alcohol

			Microbial metabolism in diverse environments<br>Inflammatory mediator regulation of TRP channels<br>Alcoholic liver disease<br>Alcoholism<br>Chemical carcinogenesis – reactive oxygen species	
13	Blood	Gentisate	Tyrosine metabolism<br>Benzoate degradation<br>Naphthalene degradation<br>Metabolic pathways<br>Microbial metabolism in diverse environments	Organic acids
14	Blood	Glutamate	Cyanoamino acid	Protein
15	Blood<br>Urine	Glycocholate	Primary bile acid biosynthesis<br>Secondary bile acid biosynthesis<br>Metabolic pathways<br>Bile secretion<br>Cholesterol metabolism	Fat
16	Blood<br>Urine	Glycine	Primary bile acid<br>Purine<br>Glycine, serine and threonine<br>Lysine<br>Phosphonate and phosphinate<br>Cyanoamino acid<br>Glutathione<br>Glyoxylate and dicarboxylate<br>One carbon pool by folate<br>Methane<br>Thiamine<br>Lipoic<br>Porphyrin<br>Aminoacyl –tRNA<br>Biosynthesis of plant secondary metabolites<br>Biosynthesis of secondary metabolites<br>Carbon<br>Biosynthesis of amino acids<br>Biosynthesis of cofactors<br>Vancomycin resistance<br>ABC transporters	Protein

			Biofilm restoration – Escherichia coli<br>Neuroactive ligand – receptor interaction<br>Synaptic vesicle cycle<br>Protein digestion and absorption<br>Mineral absorption<br>Central carbon metabolism in cancer	
17	Urine	Hippurate		Aromatic
18	Blood<br>Urine	Histidine	Histidine<br>Staurosporine biosynthesis<br>Beta-Alanine<br>D-amino acid<br>Aminoacyl – tRNA biosynthesis<br>Biosynthesis of plant secondary metabolites<br>Biosynthesis of alkaloids derived from histidine and purine<br>Biosynthesis of secondary metabolites<br>Biosynthesis of amino acids<br>ABC transporters<br>Protein digestion and absorption<br>Central carbon metabolism in cancer	Protein
19	Blood	Isobutyrate	Degradation of aromatic compounds<br>Protein digestion and absorption	Protein
20	Blood	Isoleucine	Valine, leucine and isoleucine degradation<br>Valine, leucine and isoleucine biosynthesis<br>Cyanoamino acid<br>Tropane, piperidine and pyridine alkaloid biosynthesis<br>Glucosinolate biosynthesis<br>Aminoacyl – tRNA biosynthesis<br>Biosynthesis of plant secondary metabolites<br>Biosynthesis of alkaloids derived from	Protein

			ornithine, lysine and nicotinic acids<br>Biosynthesis of secondary metabolites<br>2-Oxocarboxylic acid<br>Biosynthesis of amino acids<br>ABC transporters<br>Protein digestion and absorption<br>Mineral absorption<br>Shigellosis<br>Central carbon metabolism	
21	Blood	Isovalerate	Biosynthesis of alkaloids derived from histidine and purine<br>Biosynthesis of secondary metabolites<br>Protein digestion and absorption	Protein
22	Blood<br>Urine	Lactate	Glycolysis / Glucogenesis<br>Fructose and mannose metabolism<br>Pyruvate metabolism<br>Propanoate metabolism<br>Styrene degradation<br>Metabolic pathways<br>Biosynthesis of secondary metabolites<br>Microbial metabolism in diverse environments<br>cAMP signalling pathway<br>HIF – 1 signalling pathway<br>Efferocytosis<br>Glucagon signalling pathway<br>Central carbon metabolism in cancer	Carbohydrate
23	Blood	Lactose	Galactose metabolism<br>ABC transporters<br>Phosphotransferase system (PTS)<br>Carbohydrate digestion and absorption	Carbohydrate
24	Blood<br>Urine	Leucine	Valine, leucine and isoleucine degradation<br>Valine, leucine and isoleucine biosynthesis<br>Glucosinolate biosynthesis	Protein

			Aminoacyl-tRNA biosynthesis<br>Biosynthesis of plant secondary metabolites<br>Biosynthesis of alkaloids derived from histidine and purine<br>Biosynthesis of secondary metabolites<br>2-Oxocarboxylic acid<br>Biosynthesis of amino acids<br>ABC transporters<br>Protein digestion and absorption<br>Mineral absorption<br>Shigellosis<br>Central carbon metabolism	
25	Blood	Lysine	Lysine biosynthesis<br>Lysine degradation<br>D-Amino acid<br>Biotin metabolism<br>Tropane, piperidine and pyridine alkaloid biosynthesis<br>Aminoacyl – tRNA biosynthesis<br>Biosynthesis of secondary metabolites<br>Biosynthesis of alkaloids derived from ornithine, lysine and nicotinic acids<br>2-Oxocarboxylic acid<br>Biosynthesis of amino acids<br>ABC transporters<br>Protein digestion and absorption	Protein
26	Blood	Maleate	Tyrosine metabolism<br>Butanoate metabolism<br>Nicotinate and nicotinamide metabolism<br>Microbial metabolism in diverse environments	Organic acids
27	Blood	Maltose	Starch and sucrose metabolism<br>Metabolic pathways<br>Biosynthesis of secondary metabolites<br>ABC transporters<br>Bacterial chemotaxis	Carbohydrate

			Phosphotransferase system (PTS)<br>Taste transduction<br>Carbohydrate digestion and absorption	
28	Blood<br>Urine	N-Phenylacetylglycine	Phenylalanine<br>Glycine	Protein
29	Blood	Ornithine	D-amino acid metabolism<br>Biosynthesis of various secondary metabolites<br>Metabolic pathways<br>Biosynthesis of secondary metabolites	Protein
30	Blood	Pantothenate	beta-Alanine metabolism<br>Pantothenate and CoA biosynthesis<br>Metabolic pathways<br>Biosynthesis of secondary metabolites<br>Biosynthesis of cofactors<br>Vitamin digestion and absorption	Vitamin
31	Blood<br>Urine	Proline	Arginine and proline metabolism<br>Carbapenem biosynthesis<br>Prodigiosin biosynthesis<br>Novobiocin biosynthesis<br>Staurosporine biosynthesis<br>D-Amino acid metabolism<br>Aminoacyl-tRNA biosynthesis<br>Biosynthesis of secondary metabolites<br>Biosynthesis of amino acids<br>ABC transporters<br>Protein digestion and absorption<br>Mineral absorption<br>Central carbon metabolism in cancer	Protein
32	Urine	Pyruvate	Glycolysis / Gluconeogenesis<br>Citrate cycle (TCA cycle)<br>Pentose phosphate pathway	Carbohydrate

			Pentose and glucuronate interconversions<br>Ascorbate and aldarate metabolism<br>Alanine, aspartate and glutamate metabolism<br>Glycine, serine and threonine metabolism<br>Monobactam biosynthesis<br>Cysteine and methionine metabolism<br>Valine, leucine and isoleucine biosynthesis<br>Arginine and proline metabolism<br>Tyrosine metabolism<br>Phenylalanine metabolism<br>Benzoate degradation<br>Taurine and hypotaurine metabolism<br>Phosphonate and phosphinate metabolism<br>D-Amino acid metabolism<br>Pyruvate metabolism<br>Dioxin degradation<br>Xylene degradation<br>Glyoxylate and dicarboxylate metabolism<br>Butanoate metabolism<br>C5-Branched dibasic acid metabolism<br>Methane metabolism<br>Carbon fixation by Calvin cycle<br>Other carbon fixation pathways<br>Thiamine metabolism<br>Nicotinate and nicotinamide metabolism<br>Pantothenate and CoA biosynthesis<br>Lipoic acid metabolism<br>Terpenoid backbone biosynthesis<br>Biosynthesis of various other secondary metabolites	

			Biosynthesis of various antibiotics<br>Biosynthesis of plant secondary metabolites<br>Biosynthesis of phenylpropanoids<br>Biosynthesis of terpenoids and steroids<br>Biosynthesis of alkaloids derived from shikimate pathway<br>Biosynthesis of alkaloids derived from ornithine, lysine and nicotinic acid<br>Biosynthesis of alkaloids derived from histidine and purine<br>Biosynthesis of alkaloids derived from terpenoid and polyketide<br>Biosynthesis of plant hormones<br>Metabolic pathways<br>Biosynthesis of secondary metabolites<br>Microbial metabolism in diverse environments<br>Carbon metabolism<br>2-Oxocarboxylic acid metabolism<br>Degradation of aromatic compounds<br>Biosynthesis of amino acids<br>Biosynthesis of cofactors<br>Vancomycin resistance<br>Two-component system<br>Phosphotransferase system (PTS)<br>HIF-1 signaling pathway<br>AMPK signaling pathway<br>Insulin secretion<br>Glucagon signaling pathway<br>Type II diabetes mellitus<br>Insulin resistance<br>Central carbon metabolism in cancer	

			Diabetic cardiomyopathy	
33	Blood<br>Urine	Succinate	Citrate cycle (TCA cycle)<br>Oxidative phosphorylation<br>Alanine, aspartate and glutamate metabolism<br>Lysine degradation<br>Tyrosine metabolism<br>Phenylalanine metabolism<br>Chlorocyclohexane and chlorobenzene degradation<br>Pyruvate metabolism<br>Glyoxylate and dicarboxylate metabolism<br>Propanoate metabolism<br>Butanoate metabolism<br>Other carbon fixation pathways<br>Nicotinate and nicotinamide metabolism<br>Sulphur metabolism<br>Biosynthesis of plant secondary metabolites<br>Biosynthesis of phenylpropanoids<br>Biosynthesis of terpenoids and steroids<br>Biosynthesis of alkaloids derived from shikimate pathway<br>Biosynthesis of alkaloids derived from ornithine, lysine and nicotinic acid<br>Biosynthesis of alkaloids derived from histidine and purine<br>Biosynthesis of alkaloids derived from terpenoid and polyketide<br>Biosynthesis of plant hormones<br>Metabolic pathways<br>Biosynthesis of secondary metabolites<br>Microbial metabolism in diverse environments<br>Carbon metabolism	Carbohydrate

			Degradation of aromatic compounds<br>Two-component system<br>cAMP signaling pathway<br>GABAergic synapse<br>Glucagon signaling pathway<br>Central carbon metabolism in cancer	
34	Blood<br>Urine	Tyrosine	Ubiquinone and other terpenoid-quinone biosynthesis<br>Monobactam biosynthesis<br>Tyrosine metabolism<br>Phenylalanine metabolism<br>Phenylalanine, tyrosine and tryptophan biosynthesis<br>Novobiocin biosynthesis<br>Cyanoamino acid metabolism<br>Methane metabolism<br>Thiamine metabolism<br>Phenylpropanoid biosynthesis<br>Isoquinoline alkaloid biosynthesis<br>Betalain biosynthesis<br>Glucosinolate biosynthesis<br>Aminoacyl-tRNA biosynthesis<br>Biosynthesis of various other secondary metabolites<br>Biosynthesis of various antibiotics<br>Biosynthesis of various plant secondary metabolites<br>Biosynthesis of vancomycin group antibiotics<br>Biosynthesis of enediyne antibiotics<br>Biosynthesis of plant secondary metabolites<br>Biosynthesis of alkaloids derived from shikimate pathway	Protein

			Biosynthesis of secondary metabolites<br>2-Oxocarboxylic acid metabolism<br>Biosynthesis of amino acids<br>Biosynthesis of cofactors<br>Dopaminergic synapse<br>Melanogenesis<br>Prolactin signaling pathway<br>Protein digestion and absorption<br>Parkinson disease<br>Cocaine addiction<br>Amphetamine addiction<br>Alcoholism<br>Central carbon metabolism in cancer	

Supplementary Table S 3.

Nutritional composition of the three formulated treatment diets.

	Feed treatment diets		
	SIL	BG	TN
DM (g/kg fresh)	259.61	273.12	261.16
Ash (g/kg DM)	89.94	86.25	88.20
OM (g/kg DM)	910.06	913.75	911.80
ADF (g/kg DM)	347.87	339.02	356.269
NDF (g/kg DM)	543.74	511.99	537.10
EE (g/kg DM)	43.56	39.07	42.44
N (g/kg DM)	24.77	26.75	27.25
CP (g/kg DM)	154.83	167.18	170.28
GE (MJ/kg DM)	19.50	19.63	19.63
ME (MJ/kg DM)	10.78	10.69	11.09
CT (g/kg DM)	nd	13.46	0.86
CT (% DM)	nd	1.35	0.09

SIL, Silage control; BG, Salix. Beagle; TN, Salix Terra Nova; EE, ether extract; N, nitrogen; GE, gross energy; ME, metabolisable energy; CT, condensed tannin

Supplementary Table S 4.

Blood metabolite concentration (mmol/mL) of yearling ewes fed diets with differing condensed tannin inclusion

Row		Treatment				
		SIL	BG	TN	SEM	P-value
1	Protein					
2	Alanine	0.177	0.177	0.173	0.00524	0.929
3	Aspartate	0.0532	0.0608	0.0638	0.00210	0.0639
4	Cadaverine	0.0619	0.0725	0.0760	0.00627	0.680
5	Glutamate	0.0740	0.0714	0.0835	0.00361	0.393
6	Glycine	0.210	0.208	0.218	0.00504	0.711
7	Histidine	0.0254	0.0241	0.243	0.000941	0.693
8	Isobutyrate	0.0883^{a}	0.104^{b}	0.113^{b}	0.00326	**
9	Isoleucine	0.0889	0.0804	0.0958	0.00594	0.636
10	Isovalerate	0.00945	0.0110	0.0103	0.000585	0.613
11	Leucine	0.0912	0.0985	0.103	0.00380	0.375
12	Lysine	0.0953	0.0966	0.0895	0.00707	0.939
13	N-Phenylacetylglycine	0.0435	0.0352	0.0535	0.00343	*
14	Ornithine	0.0479	0.0449	0.0358	0.00506	0.794
15	Proline	0.218	0.228	0.222	0.00600	0.820
16	Tyrosine	0.106	0.115	0.107	0.00335	0.490
17	Carbohydrate					
18	Lactate	3.62	3.90	3.50	0.393	0.921
19	Lactose	0.101	0.116	0.132	0.0151	0.398
20	Maltose	0.0421	0.0461	0.0512	0.00283	0.492
21	Succinate	0.0626	0.0652	0.603	0.00407	0.892
22	Fat					
23	Glycocholate	0.0130	0.00807	0.0306	0.00651	0.163
24	Fatty Acid					
25	Acetate	0.606	0.574	0.532	0.0257	0.281
26	Benzoate	0.0221	0.0171	0.0158	0.00174	0.316
27	Organic Acid					
28	3-Phenylpropionate	0.0313	0.0297	0.0293	0.00166	0.874
29	Gentisate	0.0286	0.0260	0.0179	0.00226	0.170
30	Maleate	0.00155	0.00133	0.00143	0.0000742	0.562
31	Vitamin					
32	Pantothenate	0.0212	0.0173	0.0206	0.000857	0.0711

The symbol: '*','**', denote significance $P<0.05$ and 0.01, respectively. Values within a row with different superscripts differ significantly at $P < 0.05$.

Supplementary Table S 5.

Urine metabolite concentrations (mmol/mL) of yearling ewes fed diets with differing condensed tannin inclusion

		Treatment				
Row		SIL	BG	TN	SEM	P-value
1	Protein					
2	4-Hydroxyphenylacetate	0.575	0.757	0.929	0.0961	0.441
3	Alanine	0.121	0.109	0.111	0.0113	0.788
4	Betaine	0.923	0.842	1,02	0.133	0.981
5	Creatinine	7.59^{ab}	9.25^{a}	3.59^{b}	1.03	*
6	Glycine	0.810	1.11	0.928	0.137	0.995
7	Histidine	0.258	0.293	0.425	0.0408	0.0718
8	Isoleucine	0.122	0.112	0.113	0.0146	0.969
9	Leucine	0.479	0.112	0.113	0.0577	0.242
10	N-Phenylacetylglycine	12.7	12.3	16.7	2.34	0.574
11	Proline	1.07	1.04	1.79	0.176	0.316
12	Tyrosine	1.13	0.717	0.902	0.154	0.729
13	Lactate	4.06	3.97	2.51	1.01	0.757
14	Pyruvate	10.4	10.5	12.5	1.66	0.729
15	Succinate	0.512	0.327	0.404	0.0622	0.686
16	Fat					
17	Glycocholate	0.0657	0.0914	0.118	0.0174	0.381
18	Fatty Acid					
19	2-Hydroxybutyrate	0.681	0.692	0.808	0.0803	0.808
20	Acetate	2.45	2.20	2.28	0.301	0.716
21	Butyrate	0.503	0.514	0.677	0.0662	0.751
22	Aromatic					
23	Hippurate	67.2	67.1	70.5	9.67	0.916
24	Alcohol					
25	Ethanol	1.31	1.14	1.68	0.320	0.995

The symbol: '*' denote significance $P<0.05$. Values within a row with different superscripts differ significantly at $P < 0.05$.

45
